# How central inputs and force and velocity feedbacks determine motoneurons activity during voluntary hand movements

**DOI:** 10.1186/1471-2202-16-S1-P163

**Published:** 2015-12-18

**Authors:** Alberto Mazzoni, Francesco M Petrini, Jacopo Rigosa, Marco Capogrosso, Stanisa Raspopovic, Silvestro Micera

**Affiliations:** 1 The BioRobotics Institute, Scuola Superiore Sant'Anna, Pisa, 56026, Italy; 2 Bertarelli Foundation Chair in Translational NeuroEngineering, Institute of Bioengineering, School of Engineering, Ecole Polytechnique Federale de Lausanne, Lausanne, Switzerland; 3 Center for Neuroprosthetics, Ecole Polytechnique Federale de Lausanne, Lausanne, Switzerland

## 

Human hand motion is the result of a complex interplay of motoneurons dynamics, central inputs and feedbacks from the muscles activity. A complete picture of this interplay is still missing, also due to the difficulty of recording motoneuron activity. Thanks to a novel recording method we were able to observe motoneurons spiking activity in the human median nerve during voluntary hand movements. We characterized then the neural dynamics associated to force-varying tasks and to fixed velocity tasks involving different muscles. We used these results to develop a spiking neuron model for the interpretation of the observed relationship between motoneurons firing rate and muscle activity features shedding light on the neural interactions underlying control of hand movements.

**Figure 1:**
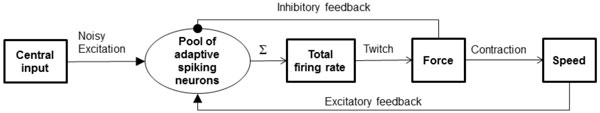
**Illustrative scheme of network structure**.

The model builds on previous studies [1,2] i) to define a common drive to motoneurons proportional to the target force and ii) to define a relationship between the motoneurons firing rate and the resulting muscular force. Starting from these works we introduced a number of modifications (Figure 1). First, we modeled single neurons as Regular Spiking Izhikevich neurons [3] rather than impulse generators, in order to take into account firing rate adaptation and to monitor relevant biological parameters. Second, we considered an unsupervised and biologically sound force feedback: instead of being determined by the difference between the prescribed target force and the actual force generated by the muscles, the feedback is determined exclusively by the resulting force. This feedback emulates the input to motoneurons sent by Ib afferent fibers. Finally, we included in the model also a specific feedback associated to the kinematic of the movements [4], analogously to the feedback contribution from Ia and II afferent fibers. We found that experimental results were reproduced only when i) central input was completely determined by the requested force; ii) force and kinematic feedback were respectively inhibitory and excitatory. Interestingly, we also found that a weak adaptation can account for a large fraction of the experimentally observed firing rate saturation at high forces even in the absence of feedback, while feedbacks are needed for the fine modulation of the outputs. Model predictions on the central input and the feedback dynamics will be tested in future experiments isolating the different components of the reflex network.
